# Mental health experiences with COVID-19 public health measures in an Alberta First Nations Community

**DOI:** 10.1186/s13033-022-00532-z

**Published:** 2022-04-29

**Authors:** Cerina Lee, Lisa A. Wozniak, Allison L. Soprovich, Vishal Sharma, Bonnie Healy, Salim Samanani, Dean T. Eurich

**Affiliations:** 1 School of Public Health, University of Alberta, 2-040 Li Ka Shing Centre for Health Research Innovation 11203-87 Avenue, Edmonton, AB T6G 2E1 Canada; 2 Blackfoot Confederacy, Calgary, AB Canada; 3 OKAKI Health Intelligence Inc., Calgary, AB Canada

**Keywords:** COVID-19, Coronavirus, Mental health, Anxiety, Depression, Public health, Epidemiology, First Nations, Population health

## Abstract

**Background:**

First Nations (FN) people of Canada experience health, social, and systemic inequities due to colonization. Consequently, COVID-19 has placed further mental health stress on people related to personal finances, employment security and worry over infection, resulting in exacerbated effects of unresolved past medical and physical traumas. This study aims to understand the experiences related to mental health in an Alberta FN community during the early stages of the pandemic.

**Methods:**

In partnership with FN leadership, the study implemented an online cross-sectional survey. Adults from a large FN community in Alberta, Canada, were asked to complete a survey, including two mental health-related screening questionnaires: (1) Generalized Anxiety Disorder-2 item; and (2) Patient Health Questionnaire-2 item. In addition, respondents could provide responses to open-ended questions about their experiences.

**Results:**

Among 106 respondents, 95 (89.6%) finished the survey; 18% of adults screened positive for depressive symptoms (score of 3 or greater) and reported difficulty following public health advice for using hand sanitizer, maintaining social distancing, or self-isolating. 21% of adults screened positive for symptoms of anxiety (score of 3 or greater) and reported difficulty maintaining social distance, self-isolating, obtaining food and clothing, or meeting other basic living requirements.

**Conclusions:**

FN communities may be disproportionately affected by COVID-19, and may experience exacerbated symptoms of anxiety, depression and overall poor mental health and well-being. Additional supports and services, including for mental health, should be considered for FN in the context of COVID-19 public health measures.

**Highlights:**

The COVID-19 pandemic has brought upon increased stress and accompanying symptoms of anxiety and depression for a First Nations community in Alberta.

Studies, such as this one, that characterize the influence of the COVID-19 pandemic on mental health among First Nations people, are urgently needed because of increasing demands on healthcare systems due to the pandemic and potential delays in the care of patients living with pre-existing mental health conditions.

There is an opportunity to capitalize on First Nations people’s experiences of post-traumatic growth proactively supporting/maintaining their well-being and possibly the development of community-based mental health interventions and supports.

**Supplementary Information:**

The online version contains supplementary material available at 10.1186/s13033-022-00532-z.

## Background

With the COVID-19 pandemic, mental health has become a rising public health concern as the crisis has generated ongoing stress in the population [21]. Internationally, the WHO Department of Mental Health and Substance Use [23] has released a series of health communication messages to support mental and psychosocial well-being for a variety of groups during the outbreak. Likewise, in Canada, the COVID-19 National Survey [14] by the Centre for Addiction and Mental Health, determined (as of August 2020) that approximately 20% of Canadians experience moderate to severe anxiety; 27.2% are binge drinking; 23% are feeling lonely and 18.7% are feeling depressed. These experiences may be further exacerbated for vulnerablilized and marginalized populations by pre-existing challenges related to social determinants of health [5].

One of the most vulnerabilized populations in Canada are First Nations people living on reserve. Prior to COVID-19, the Canadian Community Health Survey showed a higher percentage of those living on reserve reported being sad, blue or depressed for 2 weeks in a row or more [12]. Past studies and reports on mental health in First Nations peoples show that the prevalence and risk of anxiety, depression, suicide, and post-traumatic stress disorder is significantly higher than the general population [9]. Consequently, First Nations mental health is one of the top priorities listed in the Mental Health Strategy for Canada as communities continue to heal from the intergenerational impacts of colonization [13].

Today, COVID-19 has placed further mental health stress on people related to personal finances, employment security and worry over infection, resulting in exacerbated effects of unresolved past medical and physical traumas. As of August 2020, Alberta had reported the highest number of new COVID-19 cases in First Nations communities compared to other Canadian provinces and territories [8]. The objective of this descriptive study was to understand the mental health experiences of First Nations people living in a large community in Alberta during COVID-19, including public health advice outlined by the Public Health Agency of Canada and the community health services, which could be used by the community to help inform their response to COVID-19.

## Material and methods

### Design and participants

This project was driven by the expressed needs, priorities and valued outcomes of the First Nations leadership and knowledge users of which we have an established working relationship. Although our research team is trained in traditional western approaches to health services research, we have over 20 years experiences working within First Nation communities and the First Nations leadership directed all aspects of the project. In particular, the community leaders were interested in the specific assessment of public health measures around COVID-19, as opposed to the boarder impacts of COVID-19 on First Nations health and well-being. The community is well known to the researchers and after initial engagement meetings, the survey was co-created and distributed to public health leadership and front-line public health staff within the community for input. As with all our First Nations work, a research agreement was established prior to project launch outlining the roles and responsibilities and ownership of the data by the First Nations community in accordance with the FN principles of Ownership, Control, Access and Posession (OCAP). Indeed, all data has already been presented and returned to the First Nations Leadership and Knowledge users. Importantly, our authorship team includes a First Nations leader who has extensive expertise in appropriate methodology to answer the research question and relevant community context.

The First Nations community had existing infrastructure and public health support systems to monitor COVID-19, including testing, case-finding, prevention, isolation support, tailored communication, and provision of appropriate care as required, including for mental health services. Between April 24 and June 25, 2020, individuals over the age of 18 within the community were recruited to complete an online survey using several approaches and strategies, including invitations through social media (Twitter, community Facebook page) and other forms of public advertisements on the advice of First Nations leadership. Only one member per household was eligible to complete the survey as responses among family members would likely be similar with respect to attitudes towards public health advice around COVID-19.

Eligible participants who expressed interest in participating were given access to a cover letter containing relevant study information and the survey through an online portal via a unique URL. All survey information and responses were anonymous and confidentially collected in Qualtrics XM platform, which was available via cellular phones, tablets, or computers. The information collected followed OCAP principles to ensure that ownership and use of the data collected will be determined by the First Nations community leadership.

This was an unregistered, cross sectional, open survey. Items were not randomized or alternated. Adaptive questioning and logic branching were used to reduce irrelevancy and complexity of questions. The survey was 7 pages and the number of items per page varied depending on the measure (type of question). Most pages had only 2–3 items (as displayed on a single screen view) to maximize completion rates. All applicable close-ended questions had a “prefer not to answer” or “I don’t know” response option. If a question was left un-answered, the respondent was prompted to go back and select a response through the platform. Respondents were able to review and/or change their answers throughout the survey. Counting unique visitors was not applicable to this survey due to the sensitive nature of the survey questions. Thus, survey responses were completely anonymized, IP addresses were not recorded, and unique visitors were not assessed. No identification of multiple entries was used. The final question on the survey gave the choice to be entered into a draw, for a chance to win 1 of 4 $250 gift certificates, managed by the First Nations community.

### Self-reported (survey) measures

#### Demographics

Basic demographics and comorbidities (e.g. age, status, employment, income, number of household members, current medical conditions) were collected. Gender was categorized as male, female, transgender or other (with the option to specify).

### Public health advice or actions and impact of COVID-19 on basic living requirements

A matrix of questions (see Additional file 1: Appendix A) about the public health advices or actions (e.g., ability to quarantine (self-isolate), maintain physical distancing and limit contact with high-risk groups, maintain proper hand hygiene, follow travel advice) were asked. Additionally, questions about food, shelter, clothing, transportation, childcare, essential medical care and safety were asked. Where possible, questions were used or adapted from other First Nations surveys completed in the province [8]; however, many questions were co-developed specific to public health advice for COVID-19. They were not specific to mental health.

### Generalized Anxiety Disorder-2 Item

The GAD-2 questionnaire [19] is a self-administered tool for assessing for generalized anxiety disorder and anxiety-related symptoms for the past 2 weeks. Item scores range from 0 ‘not at all’ to 3 ‘nearly every day’. Using the threshold score of 3, the GAD-2 has a sensitivity of 76% and a specificity of 81% for assessing symptoms for GAD [24].

### Patient Health Questionnaire-2 Item

The PHQ-2 questionnaire is a self-administered tool for screening for depressive symptoms over the past 2 weeks [20]. Shortened from the original 9-item instrument, 2 items are scored by frequency from ‘not at all’(0) to ‘nearly every day’ (3) resulting in a score between 0–6 (higher scores representing greater depressive symptoms). A score of 3 is the cut point for further assessment for depression by a health care professional. The PHQ-2 has a reported sensitivity of 97% and a specificity of 64% for screening for major depressive symptoms. The PHQ-2 can only screen for depression and depression-related symptoms; it cannot diagnose Major Depressive Disorder or other depressive-like disorders.

### Open-ended questions

Open-ended questions were included to allow participants to expand on their survey responses and highlight their experiences with COVID-19 and public health guidelines: (1) Please tell us why it was easy or difficult for you to follow any of the advice from public health experts; (2) Please tell us about how the advice from public health experts changed your experiences, if at all; (3) What unseen benefits or “silver linings”, if any, have you experienced because of public health advice or COVID-19; and (4) Is there anything else you’d like to tell us about your experiences with public health advice about COVID-19?

## Data analysis

The data analysis was descriptive with the aim of informing the public health response within the community. All variables were reported descriptively as proportions or means (standard deviations) where appropriate. In addition, differences in reported appropriateness of the public health advice or impact of COVID-19 on basic living requirements were further explored according to baseline characteristics (e.g., age, sex, comorbidities, number of household members, employment). Differences were examined using chi-square test or Students t-test, as appropriate. No multivariate analyses were planned or conducted. All data analysis was conducted using the Qualtrics XM software [25] and Microsoft Excel, or Stata MP v15 [27]. Responses to open-ended questions were analyzed using summative content analysis [15] and managed used ATLAS.ti Version 8 [26]. We provide direct quotes documented by a participant number in parentheses (e.g., 52 represents participant 52). Each item or measure was analyzed via complete case analysis. Denominators are provided in the Results section. Time to complete the questionnaire was not assessed. No statistical corrections were needed nor completed.

## Ethics approval

The study was approved by the health research ethics board at the University of Alberta (# Pro00099663).

## Results

A total of 106 accessed and started the survey; 98 (92.5%) completed the instruments and were included in the analysis. On average, the respondents completed 90.8% of the items in the survey. Over 80% of participants were female; no significant differences were found for other demographics based on gender; no participants identified as transgender or other (Table 1). Overall, 45% of participants were over 45 years old, 53% were currently married or common law, and 60% completed post-secondary education. The number of people living together in a household was categorized, with 50% of participants living with 3–5 people. Fifty participants (47%) reported one or more existing chronic conditions. All participants were living on-reserve. Related to mental health, 10% of participants reported having an existing mental health condition. In addition, 26% of respondents stated they experienced an increase in alcohol use or other substances and 37% expressed increased concern for their safety or the safety of people with whom they interacted closely during COVID-19. These experiences were not significantly different by gender.

**Table 1:** Participant characteristics

Characteristic	n (%)			p-value
	Total sample (n = 106)	Male (n = 20)	Female (n = 86)	
Age, years				
18–30	26 (24.5)	4 (20.0)	22 (25.6)	0.3463
31–44	32 (30.2)	4 (20.0)	28 (32.6)	
45–59	35 (33.0)	10 (50.0)	25 (29.1)	
60 or older	13 (12.3)	2 (10.0)	11 (12.8)	
missing (n)	0	0	0	
Marital status				
Never married	38 (35.8)	6 (30.0)	32 (37.2)	0.5048
Married/common law	56 (52.8)	13 (65.0)	43 (50.0)	
Divorced	6 (5.7)	0 (0.0)	6 (7.0)	
Widowed	6 (5.7)	1 (5.0)	5 (5.8)	
missing (n)	0	0	0	
Education				
No formal education	0 (0.0)	0 (0.0)	0 (0.0)	0.1547
Completed grade school (grades 1–9)	6 (5.8)	3 (15.8)	3 (3.5)	
Completed high school	36 (34.6)	6 (31.6)	30 (35.3)	
Completed Trades Certificate, College, or University	57 (54.8)	10 (52.6)	47 (55.3)	
Completed Graduate education (MS or PhD)	5 (4.8)	0 (0.0)	0 (0.0)	
missing (n)	2	1	1	
Employment status immediately before COVID-19				
Full-time	45 (43.3)	9 (45.0)	36 (42.9)	0.0830
Part-time	6 (5.8)	1 (5.0)	5 (6.0)	
Self-employed	11 (10.6)	6 (30.0)	5 (6.0)	
Self-reliant or sufficient/living off the land	0 (0.0)	0 (0.0)	0 (0.0)	
Homemaker	8 (7.7)	0 (0.0)	8 (9.5)	
Not in labor force – disabled	4 (3.8)	0 (0.0)	4 (4.8)	
Retired	5 (4.8)	1 (5.0)	4 (4.8)	
Unemployed	16 (15.4)	2 (10.0)	14 (16.7)	
Other	9 (8.7)	1 (5.0)	8 (9.5)	
missing (n)	2	1	1	
Annual household income				
Less than $20,999	23 (22.1)	3 (15.0)	20 (23.8)	0.2838
$21,000-$34,999	23 (22.1)	5 (25.0)	18 (21.4)	
$35,000-$59,999	24 (23.1)	2 (10.0)	22 (26.2)	
> $60,000	22 (21.2)	7 (35.0)	15 (17.9)	
Don’t know	7 (6.7)	1 (5.0)	6 (7.1)	
Prefer not to answer	5 (4.8)	2 (10.0)	3 (3.6)	
missing (n)	2	0	2	
Living arrangement immediately before COVID-19				
Own house or condominium	52 (50.5)	12 (63.2)	40 (47.6)	0.6363
Renting, rooming house	36 (35.0)	5 (26.3)	31 (36.9)	
Staying with family or friends/Couch surfing	11 (10.7)	1 (5.3)	10 (11.9)	
Shelter	1 (1.0)	0 (0.0)	1 (1.2)	
Without a place to stay/homeless	0 (0.0)	0 (0.0)	0 (0.0)	
Prefer not to answer	3 (2.9)	1 (5.3)	2 (2.4)	
missing (n)	3	1	2	
Number of people living in the household				
1–2	26 (26.3)	5 (27.8)	21 (25.9)	0.6398
3–5	49 (49.5)	9 (50.0)	40 (49.4)	
6–8	22 (22.2)	3 (16.7)	19 (23.5)	
9 or more	2 (2.0)	1 (5.6)	1 (1.2)	
Number of existing chronic conditions				
0	48 (45.3)	9 (45.0)	39 (45.4)	0.9739
1	28 (26.4)	5 (25.0)	23 (26.7)	
2 or more	22 (20.8)	4 (20.0)	18 (20.9)	
missing (n)	8	2	6	

### Depressive symptoms

**Fig. 1:**
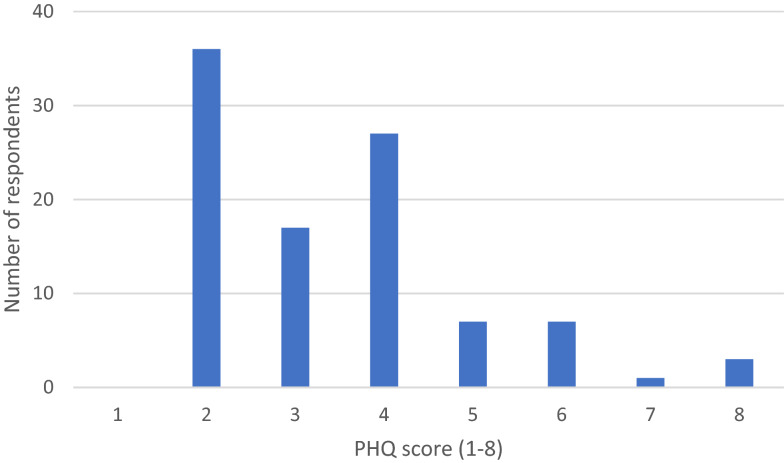
PHQ score frequency (n = 98)

Of the 98 (92%) participants who completed the PHQ-2, 18% screened positive for depressive symptoms. Scores ranged from 1–8 (Fig. 1). A higher number of females screened positive than males (p = 0.038) with 100% of males screening negative (Table 2). No significant differences were found when examining PHQ-2 scores by age, marital status, income, employment status, education, existing medical condition, or number of people in per household.

**Table 2:** Patient Health Questionniare-2 scores by gender

	n(%)			p-value
	Total sample (n = 98)	Male (n = 18)	Female (n = 80)	
Negative (< 3)	80 (81.6)	18 (100.0)	62 (77.5)	0.038
Positive (≥ 3)	18 (18.3)	0 (0.0)	18 (22.5)	
Mean		0.67	1.66	
SD		0.84	1.59	
95% CI		0.25–1.09	1.31–2.01	
Median (IQR)		0 (0–1)	2 (0–2)	

For people who screened positive for depressive symptoms, a significant number found it more difficult to follow selected public health guidelines, including the use of hand sanitizer (p = 0.040), maintaining physical distance of 2 m (p = 0.0006), self-isolating by staying home (p = 0.0190) and self-isolating by avoiding contact (p = 0.0034) (Additional file 2: Table S1). Additionally, people who screened positive for depressive symptoms found childcare ‘sometimes’ or ‘often’ challenging as a basic living requirement (p = 0.0198) (Additional file 2: Table S2).

### Anxiety symptoms

**Fig. 2:**
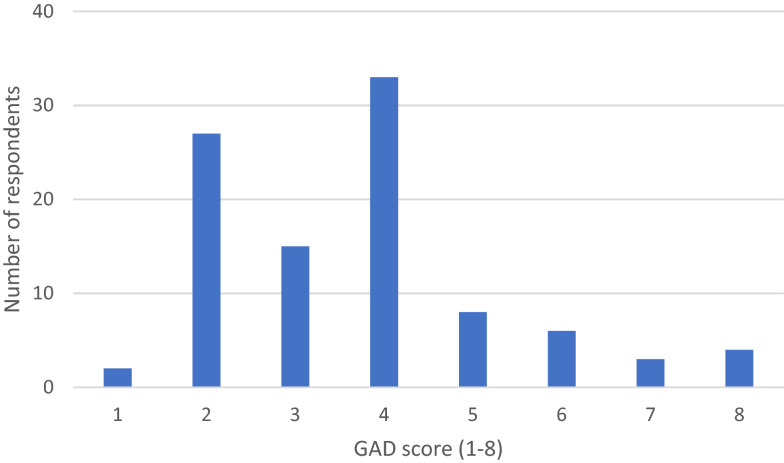
GAD score frequency (n = 98)

Of the ninety-eight (92%) participants who completed the GAD-2 screen, 21% screened positive for anxiety symptoms (score of 3 or greater). Scores ranged from 1–8 (Fig. 2). No significant differences were found between males and females (p = 0.586) (Table 3).

**Table 3:** General Anxiety Disorder-2 scores by gender

	n(%)			p-value
	Total sample (n = 98)	Male (n = 18)	Female (n = 80)	
Negative (< 3)	77 (78.6)	15 (83.3)	62 (77.5)	0.586
Positive (≥ 3)	21 (21.4)	3 (16.7)	18 (22.5)	
Mean		1.11	1.85	
SD		1.41	1.61	
95% CI		0.41–1.81	1.49–2.21	
Median (IQR)		1 (0–2)	2 (0–2)	

No significant differences were found when examining GAD-2 scores by age, marital status, household income, employment status, education level or number of people per household. However, people who reported two or more existing medical conditions were significantly more likely to screen positive for anxiety symptoms (p = 0.0397) (Table 4).

**Table 4:** General Anxiety Disorder-2 scores by number of existing medical conditions (n = 98)

	Number of existing medical conditions, n(%)			p-value
	None	1	2 or more	
Negative (< 3)	40 (51.9)	24 (31.2)	13 (16.9)	0.0397
Positive (≥ 3)	8 (38.1)	4 (19.0)	9 (42.9)	
Mean	1.42	1.61	2.5	
SD	1.44	1.64	1.65	
95% CI	1.00–1.84	0.97–2.25	1.77–3.23	
Median (IQR)	1 (0–2)	2 (0–2)	2 (2–4)	

For people who screened positive for symptoms of anxiety, a significant number found it difficult to follow selected public health guidelines, including maintaining physical distance of 2 m (p = 0.0379) and self-isolating by staying home (p = 0.0098) (Additional file 2: Table S1). Additionally, a significant number of people who screened positive for anxiety symptoms found getting food (p = 0.0055) and clothing (p = 0.0116) more challenging as basic living requirements (Additional file 2: Table S3).

### Limited awareness of mental health supports

When asked if they were aware of available mental health supports within their community, over 50% of participants responded ‘yes’, 14% responded ‘no’, and 26% responded ‘don’t know’. Responses were not significantly different by gender, age, marital status, education level, household income or living arrangement. However, awareness of mental health supports was significantly different by employment status (p = 0.0066). Fifty percent of homemakers and 60% of retired participants were not aware of available mental health supports. Conversely, 100% of participants who reported not being in the labor force due to disability and 60% of participants who reported being unemployed were aware of available mental health supports in the community. In addition, 44% of people who screened positive for depressive symptoms and 33% of people who screened positive for anxiety symptoms did not know about available mental health supports (Table 5).

**Table 5:** Mental health supports by PHQ-2 and GAD-2 scores (n = 98)

	Total sample n(%)	PHQ-2 score n(%)		GAD-2 score n(%)	
		Negative (< 3)	Positive (≥ 3)	Negative (< 3)	Positive (≥ 3)
Yes	55 (56.1)	47 (58.8)	8 (44.4)	43 (55.8)	12 (57.1)
No	14 (14.3)	12 (15.0)	2 (11.1)	12 (15.6)	2 (9.5)
Don’t know	26 (26.5)	18 (22.5)	8 (44.4)	19 (24.7)	7 (33.3)
Prefer not to answer; N/A	3 (3.0)	3 (3.4)	0	3 (3.9)	0
Mean (SD)		1.48 (1.53)		1.71 (1.59)	
Median (IQR)		1 (0–2)		2 (0–2)	
95% CI		1.17–1.79		1.39–2.03	

### Continuum of experiences of anxious symptoms and resilience

Many respondents focused on mental health and wellbeing in their responses to open-ended questions, expressing degrees of anxiousness as well as resilience to recommendations by public health experts. Respondents reported a continuum of mild to moderate experiences of anxiety symptoms from being “*more cautious*” (59) and *“more careful about my surroundings and always wash my hands now”* (24), to more severe or serious feelings of being “*worried and scared*” (15), *“lost in the fear”* (27) or “*paranoid to leave my house”* (69)*.*

Mild levels of anxiety might prompt people to protect themselves and others by adopting behaviours aligned with public health advice, like hand washing, *“only going out for essentials”* (80), *“wear PPE on a regular basis*” (30) or “*staying vigilant to [public health] protocols*” (31). At times, public health advice was reassuring to respondents, including “*Reassured me of the proper measures that we need to take into consideration before leaving the house*” (49) or “*Social distancing advice has kept our family and home safe*” (41). In some ways, people described public health advice as *“common sense”* (26, 32, 43, 87) which helped ease worries: *“…made me feel confident to follow their advice”* (26). In addition, some respondents demonstrated resilience adopting public health strategies as demonstrated by *“limiting trips and adjusting well”* (10) while recognizing, *“It was a complete lifestyle change that we had to adapt to in a very short time”* (49). In contrast, more severe levels of anxiety might impede the uptake of public health advice for some people when they believed *“nothing can help, just get through it”* (13), indicating less resilience. Indeed, some respondents had difficulty adjusting because of *“not being used to the new rules”* (56) and *“it was scary; just taking precautions”* (57). In addition, public health advice could be intimidating instead of reassuring for some people, including feelings of “*Total apprehension of others*” (50). Greater levels of anxiety could be unhealthy, and lead to more severe symptoms requiring attention:

“*It has made my father more fearful without mental therapy for elders he is getting more and more anxious*” (23).

*“It’s just made me a lot more anxious about germs more than I ever was. I noticed that my anxiety has gone up significantly”* (39).

### Personal and social benefits

Respondents also identified benefits or “silver-linings”, if any, because of COVID-19 and/or public health advice, including reassessing priorities, more leisure time, and prosocial behaviour. During this time, people reported reassessing their priorities, including *“self-examination to re-prioritize my life in a holistic way”* (4), *“learn to live a simpler life”* (29), and being *“more conscious of our actions, e.g. do we really need to go anywhere or shall we just stay home?”* (27) People also reported more leisure time to engage in meaningful personal activities including *“Clean my house. Read books I’ve wanted to read… and concentrate on a beading project…More praying”* (4). In addition, increased leisure time strengthened relationships through *“more time with family”* (23), *“Quality time spent with family has increased immensely, we sit down for meals”* (27), or *“[spending] more time with my partner, pets”* (1). Furthermore, some respondents spent their leisure time sharing their history and language or participating in the community:

*“I’m always really busy with work so this time at home enabled me to spend a lot of time with my grandkids, I taught them about our history and am teaching them to speak our language”* (63).

*“Helped with community clean up. Picking up garbage humbled me”* (4).

Some respondents reported no benefits because of COVID-19 and/or public health advice: *“nothing really”* (52), whereas others expressed both personal and social “silver-linings”.

Respondents also described other prosocial behaviours because of COVID-19 and/or public health advice, including demonstrations and acceptance of kindness and compassion:

*“People seem to be kinder and more thoughtful”* (55).

*“People being a little more compassionate”* (46).

*“I feel humbled by the care packages… Drive by parades – showing care and concern”* (65).

Furthermore, *“knowing there is help when needed”* (54) helped people feel supported during an *“unbelievable experience”* (20), which may help reduce mild anxious symptoms. Furthermore, respondents described *“tribal unity”* (37) and increased appreciation for public health: “*This pandemic made me realize how important our vulnerable population is and why we must protect them from every disease, not just coronavirus”* (21).

## Discussion

Anxiety is a normal and expected reaction to a pandemic; however, too much anxiety can be problematic and negatively affect peoples’ mental health and well-being. Our study showed that while 10% of individuals in this First Nations community reported an existing mental health condition, approximately 20% screened positive for depressive and anxiety symptoms during COVID-19 and while reacting to its associated public health measures, indicating the need for further assessment by health professionals.

The health disparities of First Nations people, including mental health, are a result of deeply rooted aspects of colonization and colonialism. The resulting oppression experienced contributes significantly towards inequities, not merely individual or community vulnerabilities. Social determinants of health such as unemployment, food security, health, social exclusion, discrimination – all play a significant role in the mental health challenges that the First Nations people face in Canada [6]. In addition, the compounding effects of these health, social and systemic inequities; discrimination and racism; isolation and inability to travel due to the lack of resources or travel restrictions, have created the ‘perfect storm’ with the potential to have immense long-term negative impacts on First Nations communities in Alberta and elsewhere.

Previous studies/reports on the First Nations peoples in Canada show consistent evidence that the intergenerational effects from the historical trauma have resulted in higher depression, suicides and other mental health disparities in this population, compared to the general population [17]. The First Nations Regional Longitudinal Health Survey (2002/2003) and the National Aboriginal Health Organization (2006) both have reported that 25.7%-34.5% of First Nations women living on reserved felt sad or depressed for two weeks or more. Likewise, individuals living off reserve also showed increased levels of depression compared to the general population (13.2% vs. 7.3%) [17]. A recent Canadian report on mental health among Indigenous people showed that more women reported symptoms consistent with moderate or severe generalized anxiety compared to men (48% and 31% respectively) during COVID-19 [8]. While our results are consistent with this finding, it is unknown why our study showed even lower anxiety in men. One possible explanation could be the strength of the community’s existing health infrastructure, which may have resulted in overall improved mental health status in both women and men or could be explained by healthy volunteer bias by males in the study. Regardless, in our study, 10% of participants reported having an existing mental health condition and 20% screened positive for depressive and anxiety symptoms.

Thus, knowing these pre-existing mental health disparities in the First Nations population, supports the inference that communities may experience higher rates of anxiety and depression during times of elevated stress (such as the COVID-19 pandemic). Approximately 50% of respondents were unaware of available mental health supports in this community. As such, more outreach efforts are needed to ensure mental health supports are known and accessible to community members.

Although there is little evidence on the self-efficacy of adult First Nations populations, studies on other vulnerabilized populations have examined the impact of self-efficacy on anxiety and depression. According to Bandura [4], self-efficacy is typically defined as one’s ability to execute behaviors and/or have control over one’s motivation and environment [4]. Research conducted by Allenden et al. [1] found self-efficacy to be a predictor of depression among older adults; those with higher self-efficacy were less likely to have depression. Additionally, for those living with chronic health conditions requiring self-care behaviours and lifestyle adjustments, like diabetes, low self-efficacy may compound with anxiety and depressive symptoms, and lead to poorer health outcomes [2, 10, 16]. In relation to the previous Influenza A pandemic in 2009, factors associated with taking preventive measures and intent to comply with government-advised preventive measures included high self-efficacy [7]. However, this does not strictly indicate that non-compliance to these measures results in lower self-efficacy in First Nations communities. As we found in our study, those experiencing symptoms of anxiety and depression may have a harder time adjusting in periods of stress, hence, following public health advice. Although we did not measure self-efficacy, the underlying construct is evident. When behaviours, thoughts, and emotions are unpredictable and outside of one’s control, such as in a pandemic, good psychological adjustment is challenging for everyone. Those with more pessimistic outlooks, feeling that bad outcomes cannot be avoided, like people with symptoms of anxiety and depression, this emotional distress may lead people to require professional help [22].

While approximately one-fifth of respondents reported anxiety and depressive symptoms, many respondents focused on positive consequences during COVID-19 and its associated public health measures, including reassessing priorities, more leisure time to pursue meaningful activities and strengthen relationships, and prosocial behaviours, such as kindness and compassion and a sense of community. Indeed, our study showed signs/indications of “post-traumatic growth” for some people in this community. Post-traumatic growth is positive changes, including increased appreciation for life, changed priorities, more meaningful relationships, and increased personal meaning, that result from highly challenging life situations [28]*.* There is an opportunity to capitalize on people’s experiences of post-traumatic growth proactively supporting/maintaining their well-being and possibly the development of community-based mental health interventions and supports. This, in turn, may alleviate some of the mild anxious and depressive feelings felt by individuals and contribute towards overall community resilience.

Overall, this study provides insight about mental health experiences among people in a First Nations community during COVID-19 intended to inform the First Nations leadership and health managers who directed the study within the community and similar communities. These health leaders and policy makers can utilize this study to inform effective health messaging and service delivery. A major strength of this study is that it is one of the first studies on the mental health experiences of people in a First Nations community in relation to COVID-19. Studies, such as this one, that characterize the influence of the COVID-19 pandemic on mental health among First Nations people, are urgently needed because of increasing demands on healthcare systems due to the pandemic and potential delays in the care of patients living with pre-existing mental health conditions.

## Limitations

The 106 participants in this study represent only a small subset of the community and these participants may not be representative. This community had sufficient health infrastructure and supports in place, so it may have been easier for participants to access health services and to follow the public health guidelines. As mentioned, this community had a pre-existing robust health system, meaning that results of this study may not be generalizable to other First Nations communities where necessary supports are not available. Healthy volunteer bias may be likely, which may inflate higher education-level estimates and underestimate anxiety and/or depression scores. Indeed, the houseless, marginalized, and very low-income individuals are much less likely to participate. Indeed, our sample contained more female participants than males and interestingly no males indicated issues with anxiety and/or depression. Although this may be true, it is more likely that males systematically under-reported their state of mental health and /or only those males who had good mental health participated (i.e., health volunteer bias). In addition, our sample also had high rates of post-secondary education compared to national averages. It is possible those with high education were more comfortable providing their information and opinions within the community and therefore participated (i.e., potential biases around health literacy). Moreover, First Nation communities in Alberta, and elsewhere, are highly heterogenous and these particular findings may only partially apply to other communities. Although it is unclear how these issues around the context of the participants impacted the results, the potential for biases exist (e.g., healthy volunteer bias) and interpretation of the findings within this context is necessary. It is important to note that the project, although in collaboration with First Nations communities, is still designed and primarily rooted in western approaches to research. We fully acknowledge the value of both this and different types of health research, including those informed by and/or rooted in Indigenous approaches, specifically in the field of psychology and mental health. Although the PHQ-2 and GAD-2 have been extensively employed in Indigenous people [18] and are well validated in other populations, we are unaware of validation studies of either instrument among Indigenous people per se. Thus, further research is required to validate the GAD-2 and PHQ-2 in First Nations communities [3, 11].

## Conclusion

With the persisting health, social and systemic inequities for First Nations people, the COVID-19 pandemic has brought upon increased stress and accompanying symptoms of anxiety and depression, as we found for people in this First Nations community. Additional individual and community supports and services, including for mental health, should be considered for First Nations in the context of COVID-19 public health measures, to foster overall resilience.

## Supplementary Information

**Additional file 1: Appendix A.** Public Health Advice on COVID-19 and First Nations Survey.

**Additional file 2: Table S1. **Ease of following public health guidelines by PHQ-2 and GAD-2 score. **Table S2. **Struggles meeting basic living requirements for people with depressive symptoms, PHQ-2 score (≥3) (n=18). **Table S3. **Struggles meeting basic living requirements for people with symptoms of anxiety, GAD-2 score (≥3) (n=21).

## Data Availability

All data and materials are available by written request to the corresponding author, DTE.

## Abbreviations

COVID-19: Coronavirus disease 2019
FN: First Nations
GAD-2: Generalized Anxiety Disorder 2-item
OCAP: Ownership, control, access and possession
PHQ-2: Patient Health Questionnaire 2-item

## Declarations

### Transparency declaration

DTE affirms that the manuscript is an honest, accurate, and transparent account of the study being reported; that no important aspects of the study have been omitted; and that any discrepancies from the study as planned (and if relevant) have been explained.
