# Supplementary material for: Mental health experiences with COVID-19 public health measures in an Alberta First Nations Community
Source: Int J Ment Health Syst. 2022 Apr 29;16:22. doi: 10.1186/s13033-022-00532-z (PMC9051493; doi:10.1186/s13033-022-00532-z)
Supplement: Supplementary file 1 — Additional file 1: Appendix A. Public Health Advice on COVID-19 and First Nations Survey. [file 13033_2022_532_MOESM1_ESM.docx]

**Additional file 1**

**Appendix A: Public Health Advice on COVID-19 and First Nations Survey**

**Public Health Advice on COVID-19 and First Nations**

**Survey**

Thank you for participating in our study by answering the following survey. Below is some information to help you answer this survey.

This confidential survey is about the appropriateness of public health advice for COVID-19 for First Nations and Metis people in Alberta.

There are no right or wrong answers to any of the questions.

Please read the questions carefully and answer each one according to what is true for you. Please answer each question to the best of your ability and please do not skip any questions.

**Section A: About You**

***We would like to know more about you***.

**A1. Are you**:

🞎 Male

🞎 Female

🞎 Transgender

🞎 If these categories do not accurately reflect how you identify yourself, please use this space to write in your response: __________________________

🞎 Prefer not to answer

**A2. What is your age?**

🞎 18-30

🞎 31-44

🞎 45-59

🞎 60 or older

**A3. Which of the following best describes your ethnic background?**

🞎 First Nations Status from Treaty area in Alberta

🞎 First Nations Status from another province

🞎 First Nations non-status

🞎 Metis

🞎 Other; please specify: ___________

🞎 Prefer not to answer

**A4. What is your marital status?**

🞎 Never married

🞎 Married / common law

🞎 Divorced

🞎 Widowed

**A5. What is the highest level of education you have completed?**

🞎 No formal schooling

🞎 Completed grade school (grades 1-9)

🞎 Completed high school

🞎 Completed College or University

🞎 Completed Graduate education (MS or PhD)

**A6. What are the first 3 letters of the postal code where you are living/staying** (for example, if your postal code was T7J 3W1, you would write T7J):

🞎 First 3 letters: _ _ _

🞎 Not applicable

🞎 Prefer not to answer

***For the following questions, please think about the time right before COVID-19 as of March 1, 2020***.

**A8. What was your employment status immediately before to COVID-19?**

🞎 Homemaker

🞎 Full-time employee

🞎 Part-time employee

🞎 Self employed

🞎 Self-reliant or sufficient / living off the land

🞎 Not in labor force-disabled

🞎 Retired

🞎 Unemployed

🞎 Others; please specify: _____________

**A9. Which of the following categories best describes your total *annual* household income immediately before to COVID-19?** (This should include income, before taxes, from all sources, wages, rent from properties, social security, disability benefits, help from relatives and so on…)

🞎 Less than $20,999

🞎 $21,000 to 34,999

🞎 $35,000 to $59,999

🞎 Greater than $60,000

🞎 Don’t know

🞎 Refuse to answer

**A10. Which best describes your living arrangement immediately before to COVID-19?**

🞎 Own house or condominium

🞎 Renting, rooming house

🞎 Staying with family or friends / Couch surfing

🞎 Shelter

🞎 Without a place to stay / homeless

🞎 Prefer not to answer

**A11. How many people (including children, adults, grandparents, friends, etc.) were living with you or staying with you in the same place immediately before to COVID-19?** ___ (number)

**Section B: Your experiences**

*“****Public health****is defined as the organized efforts of society to keep people healthy and prevent injury, illness and premature death. It is a combination of programs, services and policies that protect and promote the****health****of all****Canadians****. Throughout Canada, this work is led by the Public Health Agency of Canada.*

*During COVID-19, the Public Health Agency of Canada has given specific advice to Canadians. You may have heard this advice from the Public Health Agency of Canada directly, or through local or regional organizations, like your local Health Services. We would like to know how appropriate this advice has been for you in your current situation.*

**B1. Please tell us how easy or difficult it has been for you to follow the advice by public health experts.**

- Proper hand washing often with soap and water for at least 20 seconds, especially after using the washroom and when preparing food
- Use alcohol-based hand sanitizer if soap and water are not available
- Avoiding crowded places and non-essential gatherings
- Limiting contact with people at higher risk like older adults and those in poor health
- Keeping a distance of at least 2 arms-length (approximately 2 metres or a hockey stick) from others
- Self-isolate by staying at home and monitor yourself for symptoms
- Self-isolate by avoiding contact with others to help prevent transmission of the virus
- Follow travel advice including avoiding all non-essential travel

| 🞎 Very easy | 🞎 Somewhat easy | 🞎 Somewhat difficult | 🞎 Very difficult | 🞎 Prefer not to answer |
| --- | --- | --- | --- | --- |

Please tell us why it was easy or difficult for you to follow any of the advice from public health experts: __________________________________________________________

Have you considered or are you taking **other advice or action** suggested from other sources (such as social media like Twitter or Facebook, news sites) that may not been endorse by public health agencies?

| 🞎 Yes | 🞎 No | 🞎 Not Sure | 🞎 Prefer not to answer |
| --- | --- | --- | --- |

How confident are you in the advice you’re getting from public health experts.

| 🞎 Very confident | 🞎 Somewhat confident | 🞎 Somewhat not confident | 🞎 Not confident at all | 🞎 Prefer not to answer |
| --- | --- | --- | --- | --- |

Because of public health advice about COVID-19, have you struggled to meet the following basic living requirements? (have to borrow money, miss bill payments to satisfy your basic living needs)

- Food
- Shelter
- Clothing
- Transportation
- Child care
- Essential medical care (medications)

| 🞎 Not at all | 🞎 Sometimes | 🞎 Often | 🞎 Always | 🞎 Don’t know | 🞎 Not applicable | 🞎 Prefer not to answer |
| --- | --- | --- | --- | --- | --- | --- |

Because of public health advice about COVID-19, have you experienced any of the following?

- Increased use of alcohol or other substances
- Increased concerns for your safety or for the safety of people with whom you interact closely (for example, increased violence)

| 🞎 Yes | 🞎 No | 🞎 Not Sure | 🞎 Prefer not to answer |
| --- | --- | --- | --- |

Please tell us about how the advice from public health experts changed your experiences, if at all: _____________________________________________

**Section C: Your Health**

***We would like to know about your general health and the health of other people you interact with closely. Please answer these questions based on your current situation today.***

Has a doctor ever told you that you have (had) any of the following conditions?

*(Check all that apply)*

|  | **Yes** | **No** | **I don’t know** |
| --- | --- | --- | --- |
| Problems that make it hard for you to breathe (example, Asthma) | 🞎 | 🞎 | 🞎 |
| Cancer | 🞎 | 🞎 | 🞎 |
| Heart problems (history of heart attack) | 🞎 | 🞎 | 🞎 |
| Stroke | 🞎 | 🞎 | 🞎 |
| Diabetes | 🞎 | 🞎 | 🞎 |
| Problems with your immune system (example, Crohn’s) | 🞎 | 🞎 | 🞎 |
| Kidney Disease | 🞎 | 🞎 | 🞎 |
| Liver Disease | 🞎 | 🞎 | 🞎 |
| Mental illness | 🞎 | 🞎 | 🞎 |

Please tell us more about your emotional health. Over the last 2 weeks, how often have you been bothered by any of the following problems?

|  | **Not at all** | **Several days** | **More than half the days** | **Nearly every day** |
| --- | --- | --- | --- | --- |
| Little interest or pleasure in doing things | 🞎 | 🞎 | 🞎 | 🞎 |
| Feeling down, depressed, or hopeless | 🞎 | 🞎 | 🞎 | 🞎 |
| Feeling nervous, anxious, or on edge | 🞎 | 🞎 | 🞎 | 🞎 |
| Not being able to stop or control worrying | 🞎 | 🞎 | 🞎 | 🞎 |

Did you feel Mental Health supports were available to you?

| 🞎 Yes | 🞎 No | 🞎 Don’t know | 🞎 Not applicable | 🞎 Prefer not to answer |
| --- | --- | --- | --- | --- |

What unseen benefits or “silver linings”, if any, have you experienced because of public health advice or COVID-19? ______________________________

__________________________________________________________________________________________________________________________________________________________________________________________________________________

Is there anything else you’d like to tell us about your experiences with public health advice about COVID-19? __________________________________________ __________________________________________________________________________________________________________________________________________________________________________________________________________________

Thank you for completing this survey.
