# Supplementary material for: Mental health experiences with COVID-19 public health measures in an Alberta First Nations Community
Source: Int J Ment Health Syst. 2022 Apr 29;16:22. doi: 10.1186/s13033-022-00532-z (PMC9051493; doi:10.1186/s13033-022-00532-z)
Supplement: Supplementary file 2 — Additional file 2: Table S1. Ease of following public health guidelines by PHQ-2 and GAD-2 score. Table S2. Struggles meeting basic living requirements for people with depressive symptoms, PHQ-2 score (≥3) (n=18). Table S3. Struggles meeting basic living requirements for people with symptoms of anxiety, GAD-2 score (≥3) (n=21). [file 13033_2022_532_MOESM2_ESM.docx]

**Additional file 2 Tables**

**Additional file 2: Table S1 – Ease of following public health guidelines by PHQ-2 and GAD-2 score**

| Guideline | PHQ-2 score  n(%) | | p-value | GAD-2 score  (%) | | p-value |
| --- | --- | --- | --- | --- | --- | --- |
|  | Negative (<3) | Positive (≥3) |  | Negative (<3) | Positive (≥3) |  |
| Proper handwashing  Easy  Difficult | 79 (98.8)  1 (1.2) | 17 (94.4)  1 (5.6) | 0.2431 | 75 (97.4)  2 (2.6) | 21 (100)  0 | 0.4556 |
| Alcohol-based hand sanitizer  Easy  Difficult | 68 (85.0)  12 (15.0) | 12 (70.6)  5 (29.4) | 0.0401 | 65 (84.4)  12 (15.6) | 15 (75.0)  5 (25.0) | 0.0978 |
| Avoiding crowded places and non-essential gatherings  Easy  Difficult | 68 (85.0)  12 (15.0) | 12 (66.7)  6 (33.3) | 0.0695 | 63 (81.8)  14 (18.1) | 17 (81.0)  4 (19.0) | 0.9277 |
| Limiting contact with people at higher risk  Easy  Difficult | 65 (81.3)  15 (18.8) | 12 (70.6)  5 (29.4) | 0.0662 | 63 (82.9)  13 (17.1) | 14 (66.7)  7 (33.3) | 0.2296 |
| Keeping a distance of 2m from others  Easy  Difficult | 67 (83.8(  13 (16.3) | 8 (47.0)  9 (52.9) | 0.0006 | 63 (82.9)  13 (17.1) | 12 (57.1)  9 (42.9) | 0.0379 |
| Self-isolate by staying home and monitoring for symptoms  Easy  Difficult  Prefer not to answer | 72 (91.1)  7 (8.9)  0 | 12 (66.7)  5 (27.8)  1 (5.6) | 0.0190 | 70 (92.1)  5 (6.6)  1 (1.3) | 14 (66.7)  7 (33.3)  0 | 0.0098 |
| Self-isolate by avoiding contact with others  Easy  Difficult  Prefer not to answer | 70 (89.7)  6 (7.7)  2 (2.6) | 10 (58.8)  7 (41.2)  0 | 0.0034 | 66 (89.2)  7 (9.5)  1 (1.4) | 14 (66.7)  6 (28.6)  1 (4.8) | 0.0646 |
| Follow travel advice  Easy  Difficult | 69 (87.3)  10 (12.7) | 13 (76.5)  4 (23.5) | 0.2655 | 65 (86.7)  10 (13.3) | 17 (80.9)  4 (19.0) | 0.6086 |
| missing (n) | 2 | 5 |  | 8 | 1 |  |

**Additional file 2: Table S2 - Struggles meeting basic living requirements for people with depressive symptoms, PHQ-2 score (≥3) (n=18)**

| Living requirement | n(%) | | | | |
| --- | --- | --- | --- | --- | --- |
|  | Not at all | Sometimes | Often | Always | N/A, Don’t know, prefer not to answer |
| Food | 7 (38.9) | 7 (38.9) | 1 (5.6) | 3 (16.7) | 0 |
| Shelter | 13 (72.2) | 2 (11.1) | 2 (11.1) | 1 (5.6) | 0 |
| Getting essential medical care | 6 (33.3) | 8 (44.4) | 2 (11.1) | 2 (11.1) | 0 |
| Clothing | 5 (27.8) | 8 (44.4) | 3 (16.7) | 2 (11.1) | 0 |
| Transportation | 11 (64.8) | 4 (23.5) | 1 (5.9) | 1 (5.9) | 0 |
| Childcare* | 3 (18.8) | 4 (25.0) | 3 (18.8) | 1 (6.3) | 5 (31.3) |

*significant result, p=0.0198

**Additional file 2: Table S3 - Struggles meeting basic living requirements for people with symptoms of anxiety, GAD-2 score (≥3) (n=21)**

| Living requirement | n(%) | | | | |
| --- | --- | --- | --- | --- | --- |
|  | Not at all | Sometimes | Often | Always | N/A, Don’t know, prefer not to answer |
| Food^ | 7 (33.3) | 6 (28.5) | 5 (23.8) | 3 (14.3) | 0 |
| Shelter | 17 (81.0) | 2 (9.5) | 0 | 2 (9.5) | 0 |
| Getting essential medical care | 6 (28.6) | 9 (42.9) | 4 (19.0) | 2 (9.5) | 0 |
| Clothing* | 5 (23.8) | 7 (33.3) | 3 (14.3) | 4 (19.0) | 2 (9.5) |
| Transportation | 12 (60.0) | 4 (20.0) | 2 (10.0) | 2 (10.0) | 0 |
| Childcare | 7 (35.0) | 3 (15.0) | 1 (5.0) | 1 (5.0) | 8 (40.0) |

^significant result, p=0.0055

*significant result, p=0.0116
